# Association of Mitochondrial DNA Variations with Lung Cancer Risk in a Han Chinese Population from Southwestern China

**DOI:** 10.1371/journal.pone.0031322

**Published:** 2012-02-21

**Authors:** Shizhen Zheng, Pin Qian, Fuxiang Li, Guisheng Qian, Changzheng Wang, Guoming Wu, Qi Li, Yan Chen, Jin Li, Haining Li, Binfeng He, Fuyun Ji

**Affiliations:** 1 Institute of Human Respiratory Disease, Xinqiao Hospital, Third Military Medical University, Chongqing, China; 2 Ultrasonography Department, Xinqiao Hospital, Third Military Medical University, Chongqing, China; National Taiwan University Hospital, Taiwan

## Abstract

Mitochondrial DNA (mtDNA) is particularly susceptible to oxidative damage and mutation due to the high rate of reactive oxygen species (ROS) production and limited DNA-repair capacity in mitochondrial. Previous studies demonstrated that the increased mtDNA copy number for compensation for damage, which was associated with cigarette smoking, has been found to be associated with lung cancer risk among heavy smokers. Given that the common and “non-pathological” mtDNA variations determine differences in oxidative phosphorylation performance and ROS production, an important determinant of lung cancer risk, we hypothesize that the mtDNA variations may play roles in lung cancer risk. To test this hypothesis, we conducted a case-control study to compare the frequencies of mtDNA haplogroups and an 822 bp mtDNA deletion between 422 lung cancer patients and 504 controls. Multivariate logistic regression analysis revealed that haplogroups D and F were related to individual lung cancer resistance (OR = 0.465, 95%CI = 0.329–0.656, *p*<0.001; and OR = 0.622, 95%CI = 0.425–0.909, *p* = 0.014, respectively), while haplogroups G and M7 might be risk factors for lung cancer (OR = 3.924, 95%CI = 1.757–6.689, *p*<0.001; and OR = 2.037, 95%CI = 1.253–3.312, *p* = 0.004, respectively). Additionally, multivariate logistic regression analysis revealed that cigarette smoking was a risk factor for the 822 bp mtDNA deletion. Furthermore, the increased frequencies of the mtDNA deletion in male cigarette smoking subjects of combined cases and controls with haplogroup D indicated that the haplogroup D might be susceptible to DNA damage from external ROS caused by heavy cigarette smoking.

## Introduction

Lung cancer has replaced liver cancer to become the leading cause of cancer-related deaths in China, accounting for 22.7% of all cancer deaths [1]. The rates of morbidity and mortality continue to rise rapidly and the lung cancer patients will reach one million in 2025 if no effective control measures were taken [2]. Cigarette smoking and asbestos are the two major causes of lung cancer, however, not all of those who have been exposed to the risk factors will develop lung cancer, suggesting that other causes, including genetic susceptibility, might contribute to the individual lung cancer risk and that gene-environment interactions may exist [3]–[5].

Until now, many studies have been focused on the genetic variants of nuclear genomic DNA encoding genes to investigate the gene–environment interaction of genetic variants of genes with lung cancer risk and found that several polymorphisms of genes such as CYP2E1, CYP1A1, XRCC1, and others are associated with lung cancer risk [6]–[9]. However, very few studies have investigated the role of mitochondrial DNA (mtDNA) variations in individual susceptibility to lung cancer. Through the Krebs cycle and oxidative phosphorylation (OXPHOS), mitochondria produce both ATP to help cells survive and approximately 85% of intracellular reactive oxygen species (ROS), which can promote cellular differentiation or induce apoptosis. Human mtDNA is a circular molecule of approximately 16.5 kb, encoding 22 transfer RNAs (tRNAs), 2 ribosomal RNAs (rRNAs) and 13 respiratory chain subunits that are essential for the respiratory functions of the mitochondria [10], [11]. Prior studies have demonstrated that some mtDNA haplogroups are associated with human susceptibility to metabolic and degenerative diseases, and influence longevity and carcinogenesis in conditions where mitochondrial ROS production is thought to play a role [12]–[15]. In addition, the common and “non-pathological” mtDNA variations that define mtDNA haplogroups have been found to determine differences in OXPHOS performance and ROS production in mice and humans [16]–[19].

It has been established that mtDNA is particularly susceptible to oxidative damage and mutation due to the high rate of ROS production and limited DNA-repair capacity in mitochondria [20], [21]. Cigarette smoking is one exposure that induces oxidative stress by creating ROS within the human body [22], [23]. Chronic oxidative stress may induce mtDNA damage, leading to point mutations, insertions or deletions [24], [25]. The accumulation of oxidative damage and the resulting sequence variations in mtDNA may ultimately lead to abnormal OXPHOS in affected cells, which may play a role in the occurrence of mitochondrial-related diseases. Recently, the increased mtDNA copy number for compensation for damage has been found to be positively associated with subsequent lung cancer risk among heavy smokers [26].

Given that the common and “non-pathological” mtDNA variations that define an mtDNA haplogroup contribute to differences in OXPHOS performance and intracellular ROS production and that the level of ROS present is an important determinant of cancer risk, we hypothesize that the mtDNA variations defining some mtDNA haplogroups may play a role in susceptibility to lung cancer. To test this hypothesis, a case-control study was conducted to investigate the role of mtDNA variation in lung cancer risk in a Han Chinese population from southwestern China. In addition, the gene-environment interactions between the mtDNA variations and cumulative cigarette smoking were analyzed.

## Materials and Methods

### Study group

Patients (n = 442) with primary lung cancer diagnosed from September 2007 to December 2008 were recruited from the Institute of Human Respiratory Disease of Xinqiao Hospital. All patients were newly diagnosed, histologically confirmed and previously untreated. 504 age and sex-matched control samples were collected from individuals at the Center of Physical Examination of Xinqiao Hospital between November 2007 and December 2008. The exclusion criterion for the control group was any history of cancer. All of the subjects were unrelated at least within three generations. After explaining the purpose and procedures of the study, all participants signed a written informed consent form and completed a detailed questionnaire regarding their smoking habits. Blood samples were drawn into Na-EDTA tubes from all subjects and stored at −70°C for genomic DNA extraction. The study was approved by the Ethical Committee of Xinqiao Hospital, Third Military Medical University.

### MtDNA haplogrouping

Genomic DNA was extracted from whole blood using the QIAamp DNA Blood Mini Kit according to the manufacturer's instructions (QIAGEN, Maryland, USA). MtDNA haplogrouping was completed as described by Li [27]. Briefly, the entire mtDNA sample was amplified into 22 overlapping PCR fragments and then digested with 14 restriction endonucleases [28], [29]. A negative control was included in each PCR-restriction fragment length polymorphism (PCR-RFLP) analysis for mtDNA haplogrouping to avoid artificial contamination caused by potential sample crossover. PCR-RFLP analysis was supplemented by sequencing the hypervariable segment I (HVS-I, from positions 16,024 to 16,383, relative to the revised Cambridge Reference Sequence of mtDNA, rCRS) [30]. Additionally, selected mtDNAs representing the major lineages (including haplogroups A, B, D, F, G, M7, and M8) were completely sequenced. The mtDNA haplotypes, based on the PCR-RFLPs and HVSI sequences, were classified into haplogroups according to the phylogenetic analyses of mtDNAs and Mitomap-Phylogeny [31]–[36].

### Detection of an 822 bp deletion in mtDNA

The primers used for detection of an 822 bp mtDNA deletion were the following: mtDNA-1 (Forward): 5′-TTCGCCTACACAATTCTCCG-3′ and mtDNA-2 (reverse): 5′-ACAGATACTGCGACATAGGG-3′. PCR amplifications were performed in a total volume of 25 µL containing 200 mmol/L of each dNTP, 0.35 µmol/L of each of the forward and reverse primers, 50 mmol/L KCl, 10 mmol/L Tris-HCl (pH 9.0), 1.5 mmol/L MgCl_2_, and 1 U of Taq DNA polymerase (Promega, Shanghai, China). Cycling conditions included a single pre-denaturation step at 94°C for 4 min, followed by 39 cycles of denaturation at 94°C for 40 s, annealing at 59°C for 30 s, elongation at 72°C for 1 min, and a final incubation at 72°C for 10 min. The PCR products were visualized using electrophoresis on 1.5% agarose gels stained with ethidium bromide. Five bands containing the deletion selected randomly were cut from agarose gels and DNA was purified using a DNA Gel Extraction Kit (GENERAY, Shanghai, China). Then the purified DNA was cloned into the pMD®18-T vector according to the manufacturer's instructions (TaKaRa, Dalian, China). Twenty clones were randomly selected for subsequent sequencing. The locations of the deletion(s) boundaries were determined by alignment of the PCR product sequence with the rCRS of mtDNA [30]. In order to detect extremely low levels of the mtDNA deletion, a nested-PCR method was applied. Primers mtDNA-1 and mtDNA-2 were used for the primary PCR reaction. 1 µl of the primary PCR products was then used as a template for the secondary reaction using primers mtDNA-1 and mtDNA-3 (Reverse: 5′- GGCTTTATGACCCTGAAGTAG-3′). The PCR conditions for the secondary reactions were similar to those described for the primary PCR. The PCR products were visualized using electrophoresis on 2.0% agarose gels stained with ethidium bromide.

### Data analyses

Cigarette smoking was stratified by the median number of pack-years of combined cases and controls (1 pack-year = 20 cigarettes per day for 1 year). Cases and controls were compared by Student's *t*-test for continuous variables and Pearson's chi-square test or Fisher's exact test for categorical variables. For multiple comparisons of mtDNA haplogroups, the Bonferroni correction was used (required significance level = 0.05 per number of comparisons). To assess the independent effect of each mtDNA haplogroup, multivariate logistic regression analyses, with adjustments for possible confounding factors (age, gender and smoking habits), were performed to calculate adjusted odds ratios (ORs) and 95% confidence intervals (95% CI). Assessment of the association between mtDNA deletion and cigarette smoking was performed using a linear-by-linear association test after stratification based on cigarette consumption. All statistical analyses were performed using the Statistical Package for Social Science 15 for Windows (SPSS Inc, Chicago, IL, USA).

## Results

### Subject characteristics

In total, 442 unrelated patients and 504 unrelated controls were recruited from southwestern China for the study. No female cigarette smokers were gathered. The descriptive characteristics of the study population were given in Table 1. The median number of pack-years of combined cases and controls was utilized as the cut-point to stratify the cigarette smoking subjects. As shown in Table 1, the distribution of tumor types among the patients was as follows: adenocarcinoma, 37.33%; squamous cell carcinoma, 26.02%; other non-small cell carcinoma, 22.4%; and small cell carcinoma, 14.25%. As expected, cases smoked more cigarettes (*p*<0.001).

**Table 1:** Characteristics of the study population.

Characteristic	Controls (n = 504) (%)	Cases (n = 442) (%)	*p*-Value
Sex			
Male	392 (77.78)	358 (81.00)	0.223^a^
Female	112 (22.22)	84 (19.00)	
Age at diagnosis (years)			
<39	31 (6.15)	24 (5.43)	0.599^a^
40–49	87 (17.26)	65 (14.71)	
50–59	162 (32.14)	146 (33.03)	
60–69	131 (25.99)	132 (29.86)	
>70	93 (18.45)	75 (16.97)	
Mean age ± SD	58.43±10.52	58.78±11.18	0.622^b^
Histology			
Adenocarcinoma	-	165 (37.33)	-
Squamous cell carcinoma	-	115 (26.02)	
Other non-small cell carcinoma	-	99 (22.40)	
Small cell carcinoma	-	63 (14.25)	
Pack-years of smoking^c^			
0–30	447 (88.69)	255 (57.69)	<0.001^a^
>30	57 (11.31)	187 (42.31)	
Mean pack-year ± SD	8.63±18.43	23.95±21.90	<0.001^b^
Mean pack-year ± SD^d^	11.10±20.23	29.39±20.71	<0.001^b^

a *χ* ^2^-Test or Fisher's exact test.

b *t*-Test.

c The median number of pack years of combined cases and controls were utilized as the cut-point.

d restricted to males only.

### MtDNA haplogrouping

The mtDNA haplogrouping was completed for all cases and controls. All subjects were classified into 17 common Asian mtDNA haplogroups by PCR-RFLPs analyses and HVS I sequencing. The distribution of mtDNA haplogroups in both cases and controls was shown in Table 2. Pearson's chi-square test or Fisher's exact test revealed that, compared with controls, mtDNA haplogroups G, M7 and M8 (M8a+C+Z) were significantly higher and haplogroups D and F significantly lower among cases (*p*<0.001, *p* = 0.001, *p* = 0.003, *p*<0.001, and *p* = 0.001, respectively). When the Bonferroni correction was applied (required significance level = 0.05 per number of comparisons), haplogroup M8 could not reach the adjusted *p* value cutoff of <0.0029. Multivariate logistic regression analyses with adjustments for age, gender, and smoking revealed that, on the basis of a *p* value of <0.05, haplogroups D and F were associated with individuals' lung cancer resistance (OR = 0.465, 95% CI = 0.329–0.656, *p*<0.001; and OR = 0.622, 95% CI = 0.425–0.909, *p* = 0.014, respectively), while haplogroups G and M7 might be risk factors for lung cancer (OR = 3.924, 95% CI = 1.757–6.689, *p*<0.001; and OR = 2.037, 95% CI = 1.253–3.312, *p* = 0.004, respectively).

**Table 2:** Distribution of mtDNA haplogroups among cases and controls.

mtDNA haplogroups	Controls (n = 504) (%)	Cases (n = 442) (%)	*p* value (*X* ^2^)	Adjusted *p* value^a^	OR (95% CI)^a^
A	36 (7.14%)	38 (8.6%)	0.406	0.294	1.309 (0.791–2.167)
B	96 (19.05%)	89 (20.14%)	0.674	0.836	1.037 (0.734–1.466)
F	104 (20.63%)	55 (12.44%)	0.001	0.014	0.622 (0.425–0.909)
D	140 (27.78%)	74 (16.74%)	0.000	0.000	0.465 (0.329–0.656)
G	14 (2.78%)	35 (7.92%)	0.000	0.000	3.924 (1.757–6.689)
M7	31 (5.95%)	56 (12.67%)	0.001	0.004	2.037 (1.253–3.312)
M8(M8a+C+Z)	31 (5.95%)	51 (11.76%)	0.003	0.109	1.511 (0.912–2.505)
M9	14 (2.78%)	5 (1.13%)	0.072	0.301	0.574 (0.200–1.674)
Y1	2 (0.4%)	6 (1.36%)	0.107	0.351	2.238 (0.412–12.172)
N9a	6 (1.19%)	7 (1.58%)	0.604	0.122	2.391 (0.792–7.219)
R3	6 (1.19%)	3 (0.68%)	0.419	0.675	0.737 (0.177–3.069)
M2	4 (0.79%)	5 (1.13%)	0.594	0.779	1.222 (0.303–4.923)
M10	6 (1.19%)	5 (1.13%)	0.932	0.585	1.408 (0.412–4.812)
M13a	5 (0.99%)	6 (1.36%)	0.601	0.420	1.655 (0.487–5.627)
M11	2 (0.4%)	0 (0%)	NA	NA	NA
M	2 (0.4%)	2 (0.45%)	1.000	0.466	2.080 (0.291–14.877)
U1	5 (0.99%)	5 (1.13%)	0.932	0.822	1.162 (0.313–4.310)

*χ* ^2^-Test or Fisher's exact test. Bonferroni corrected *p*<0.05/n (n = 17).

a , ORs (95% CIs) and *p* value determined by multivariate logistic regression analysis, adjusted for age, gender, and pack-years of cigarette smoking.

### Detection of the small deletion in mtDNA

**Figure 1:**
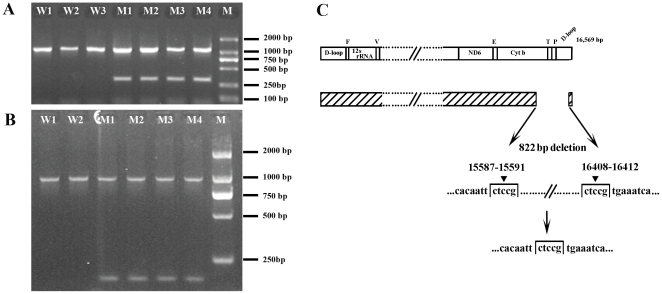
Electrophoresis for the detection of the 822 bp mtDNA deletion and the 822 bp mtDNA deletion in the schematic representation of a linearized mitochondrial genome. (A) PCR products amplified with primers mtDNA-1 and mtDNA-2. The right lane is molecular marker DL 2000. W1, W2, and W3 showing PCR products with 1129 bp from wide type mtDNA, M1, M2, M3 and M4 indicating PCR products with 1129 bp and 307 bp from mutant mtDNA. (B) PCR products amplified with primers mtDNA-1 and mtDNA-3. The right lane is molecular marker DL 2000. W1 and W2 showing PCR products with 956 bp from wide type mtDNA, M1, M2, M3 and M4 indicating PCR products with 956 bp and 134 bp from mutant mtDNA. (C) The 822 bp mtDNA deletion in the schematic representation of a linearized mitochondrial genome. Mitochondrial nucleotides were numbered according to rCRS of mtDNA [30]. Nucleotide repeats at or near the sites of cleavage are bracketed. The placement of ▾ above the bracket indicates that the exact cleavage site within the nucleotide repeat was unknown. The deleted mtDNA fragment covered from 15587–15591 nps to 16408–16412 nps. CTCCG showed the 5 bp short direct repeats at both ends of the deletion regions. The deletion was formed by cleavage within the 5 bp direct repeats.

Primers mtDNA-1 and mtDNA-2 were primarily used to detect the deletion of mtDNA. In the wide type mtDNA, the primers only yielded a product with 1129 bp. In subjects with the mtDNA deletion, the primers amplified products with 1129 bp and an aberrant product about 300 bp (Fig. 1A). DNA fragments purified from five randomly selected bands containing the deletion were cloned into pMD®18-T vector and sequenced. Sequencing data revealed that the deleted portion of mtDNA fragment was approximately 822 bp, starting between 15587–15591 nucleotide positions (nps) and ending between 16408–16412 nps (Fig. 1C). Because both ends of the deleted region have the 5 bp same nucleotides (CTCCG, 5 bp short direct repeats), the exact start and end points of the deletion could not be determined. In order to detect extremely low levels of the mtDNA deletion, a nested-PCR method was applied for all of the cases and controls. In the wide type mtDNA, the primers mtDNA-1 and mtDNA-3 only yielded a product with 956 bp. In subjects with the mtDNA deletion, the primers amplified products with 956 bp and an aberrant product with 134 bp (Fig. 1B).

### Distribution of the 822 bp mtDNA deletion among cases and controls

**Figure 2:**
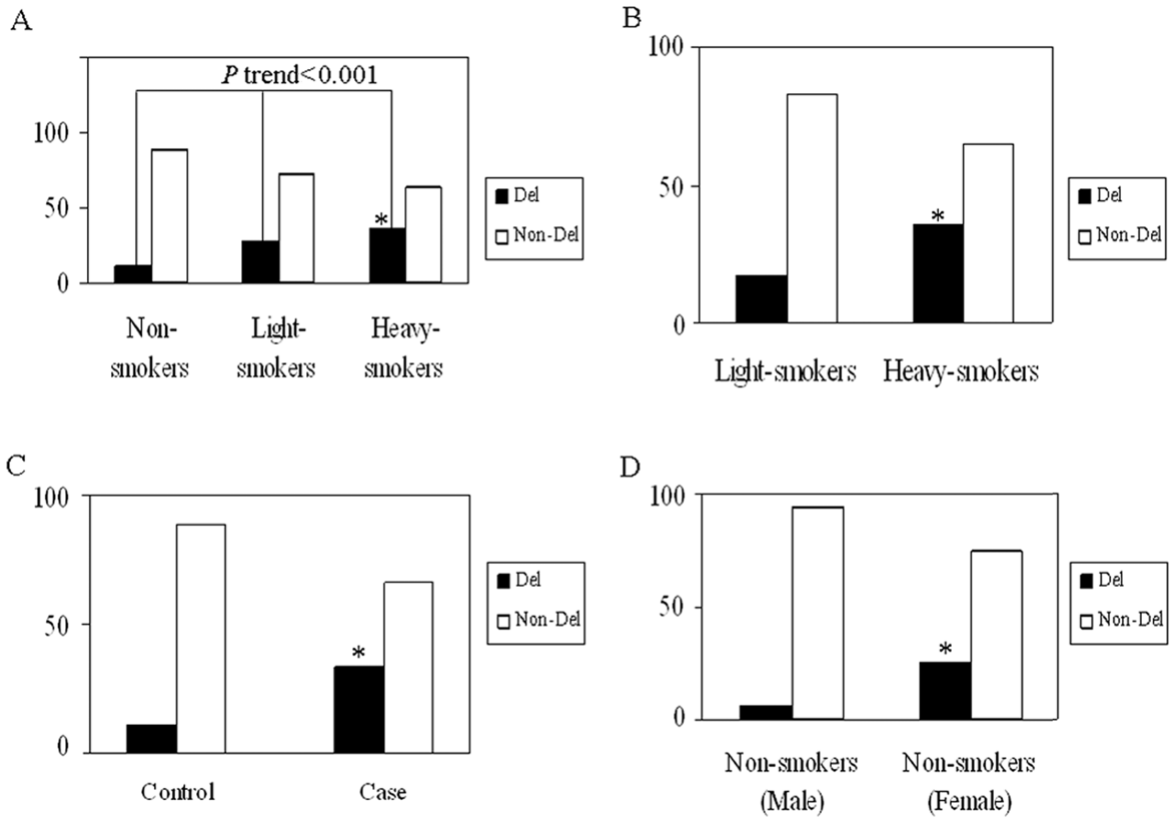
Distribution of the 822 bp mtDNA deletion among cases and controls. Del, deletion. The median number of pack-years of combined cases and controls was utilized as the cut-point to stratify the cigarette smoking subjects. The single bar depicting the proportion of individuals who had the deletion or not. * indicating *p*<0.001. (A) Distribution of the mtDNA deletion among subjects pooled from cases and controls and stratified by pack-years of cigarette smoking. Non-smokers who had no any history of cigarette smoking; Light-smokers who smoked 1–30 pack-years of smoking; Heavy-smokers who smoked >30 pack-years of smoking; *p* trend was calculated by the chi-square test for linear-by-linear association (both *p* value and *p* trend <0.001). (B) Distribution of the mtDNA deletion among light-cigarette and heavy-cigarette smoking subjects of combined cases and controls. Light-smokers who smoked 0–30 pack-years of smoking; Heavy-smokers who smoked >30 pack-years of smoking. ORs (95% CIs) and *p* value determined by multivariate logistic regression analysis, adjusted for age and gender (OR = 6.540, 95% CI = 3.247–13.174, adjusted *p* value<0.001). (C) Distribution of the mtDNA deletion among cases and controls. Compared with controls, the mtDNA deletion was significantly enriched in cases of lung cancer (*p*<0.001). ORs (95% CIs) and *p* value determined by multivariate logistic regression analysis with adjustment for age, gender and smoking habits (OR = 3.776, 95% CI = 2.662–5.355, adjusted *p*<0.001). (D) Distribution of the mtDNA deletion among non-cigarette smoking subjects pooled from cases and controls and stratified by gender. Non-smokers who had no any history of cigarette smoking. ORs (95% CIs) and *p* value determined by multivariate logistic regression analysis adjusted for age (OR = 5.814, 95% CI = 3.279–10.309, adjusted *p*<0.001).

When the subjects from both cases and controls were pooled and stratified by median number of pack-years of combined cases and controls, a subsequent linear-by-linear association test revealed an increasing trend for mtDNA deletion from non-smoking to heavy smoking groups (both *p* value and *p* trend <0.001) (Fig. 2A). The frequencies of mtDNA deletion between light-smoking and heavy-smoking subjects pooled from cases and controls and stratified by smoking habits were compared and given in Fig. 2B. Pearson's chi-square test or Fisher's exact test revealed that the deletion occurred significantly more frequently in heavy smoking subjects (*p*<0.001). Multivariate logistic regression analyses adjusted for age and gender revealed that cigarette smoking might be a risk factor for the mtDNA deletion (OR = 6.540, 95% CI = 3.247–13.174, adjusted *p*<0.001). Additionally, compared with controls, the mtDNA deletion was significantly enriched in cases of lung cancer (*p*<0.001) (Fig. 2C). Multivariate logistic regression analyses with adjustment for age, gender, and smoking habits revealed that the risk of lung cancer cases for the mtDNA deletion was 3.8 times higher than that of controls (OR = 3.776, 95% CI = 2.662–5.355, adjusted *p*<0.001). Interestingly, the occurrence of mtDNA deletion was significantly higher in female non-smoking subjects than that in male non-smoking subjects of combined cases and controls (*p*<0.001) (Fig. 2D). Multivariate logistic regression analyses adjusted for age revealed that the risk of female non-smoking subjects for the mtDNA deletion was 5.8 times higher than that of male non-smoking subjects (OR = 5.814, 95% CI = 3.279–10.309, adjusted *p*<0.001).

### Distribution of the 822 bp mtDNA deletion in major mtDNA haplogroups of combined cases and controls

The frequencies of mtDNA deletion in major mtDNA haplogroups of combined cases and controls were given in Table 3. As shown in Table 3, Pearson's chi-square test or Fisher's exact test revealed that the deletion occurred significantly more frequently in subjects with mtDNA haplogroups D (*p*<0.001). Multivariate logistic regression analyses adjusted for age, gender and smoking habits revealed that haplogroup D might be a risk factor for the 822 bp mtDNA deletion (OR = 1.906, 95% CI = 1.359–2.738, adjusted *p*<0.001). The frequencies of mtDNA deletion in major mtDNA haplogroups among male cigarette smoking subjects and male non-cigarette smoking subjects of combined cases and controls were presented in Table 4 and 5, respectively. As given in Table 4, haplogroup D was found to be a risk factor for the mtDNA deletion among male cigarette smoking subjects (OR = 2.752, 95% CI = 1.699–4.456, adjusted *p*<0.001). As shown in Table 5, the deletion was enriched in subjects with mtDNA haplogroups G among male non-cigarette smoking subjects (*p* = 0.036). Multivariate logistic regression analyses adjusted for age revealed that on the basis of a *p* value of <0.05, haplogroups G might be a risk factor for the mtDNA deletion in male non-cigarette smoking subjects of combined cases and controls (OR = 3.906, 95% CI = 1.381–12.255, adjusted *p* = 0.017).

**Table 3:** Distribution of the 822 bp mtDNA deletion in major mtDNA haplogroups among subjects pooled from cases and controls.

MtDNA haplogroups	Del (n = 206) (%)	Non-Del (n = 740) (%)	*p* value (*X* ^2^)	Adjusted *p* value^a^	OR (95% CI)^a^
A (n = 74)	15 (7.28)	59 (7.97)	0.883	0.967	0.987 (0.543–1.795)
B (n = 185)	35 (16.99)	150 (20.27)	0.322	0.302	0.805 (0.534–1.215)
M8 (n = 82)	15 (7.28)	67 (9.05)	0.486	0.295	0.729 (0.404–1.317)
G (n = 49)	11 (5.34)	38 (5.14)	0.860	0.861	1.065 (0.528–2.148)
F (n = 159)	41 (19.90)	118 (15.95)	0.206	0.090	1.416 (0.941–2.096)
D (n = 214)	65 (31.55)	149 (20.14)	<0.001	<0.001	1.906 (1.359–2.738)
M7 (n = 87)	16 (7.77)	71 (9.59)	0.496	0.189	0.677 (0.380–1.210)
Others (n = 96)	8 (3.88)	88 (11.89)	NA	NA	NA

*χ* ^2^-Test or Fisher's exact test.

For multiple comparisons, *p* value was set <0.05/2×(n−1) (n = 8).

a , ORs (95% CIs) and *p* value determined by multivariate logistic regression analysis, adjusted for age, gender and pack-years of smoking.

**Table 4:** Distribution of the 822 bp deletion of mtDNA in major mtDNA haplogroups among male cigarette smoking subjects pooled from cases and controls^a^.

MtDNA haplogroups	Del (n = 138) (%)	Non-Del (n = 290) (%)	*p* value (*X* ^2^)	Adjusted *p* value^b^	OR (95% CI)^b^
A (n = 35)	8 (5.80)	27 (9.31)	0.215	0.243	0.613(0.270–1.394)
B (n = 83)	25 (18.12)	58 (20.00)	0.645	0.519	0.842(0.498–1.422)
M8 (n = 38)	9 (6.52)	29 (10.00)	0.237	0.304	0.662(0.301–1.454)
G (n = 31)	8 (5.80)	23 (7.93)	0.426	0.551	0.774(0.334–1.794)
F (n = 65)	27 (19.57)	38 (13.10)	0.082	0.066	1.672(0.967–2.889)
D (n = 90)	46 (33.33)	44 (15.17)	<0.001	<0.001	2.752(1.699–4.456)
M7 (n = 52)	12 (8.70)	40 (13.79)	0.131	0.132	0.592(0.299–1.171)
Others (n = 34)	3 (2.17)	31 (10.69)	NA	NA	NA

Del, deletion.

For multiple comparisons, *P* value was set <0.05/2×(n−1) (n = 8).

a , Cigarette smoking subjects who had any history of cigarette smoking.

b , ORs (95% CIs) and *p* value determined by multivariate logistic regression analysis adjusted for age.

**Table 5:** Distribution of the 822 bp deletion of mtDNA in major mtDNA haplogroups among male non-cigarette smoking subjects pooled from cases and controls^a^.

MtDNA haplogroups	Del (n = 18) (%)	Non-Del (n = 304) (%)	*p* value (*X* ^2^)	Adjusted *p* value^b^	OR (95% CI)^b^
A (n = 30)	2 (11.11)	28 (9.21)	0.679	0.677	1.398 (0.290–6.736)
B (n = 61)	3 (16.67)	58 (19.08)	1.000	0.819	0.861 (0.239–3.106)
M8 (n = 24)	2 (11.11)	22 (7.24)	0.634	0.634	1.457 (0.309–6.870)
G (n = 14)	3 (16.67)	11 (3.62)	0.036	0.017	3.906 (1.381–12.255)
F (n = 61)	3 (16.67)	58 (19.08)	1.000	0.790	0.839 (0.230–3.055)
D (n = 72)	3 (16.67)	69 (22.70)	0.772	0.625	0.726 (0.202–2.613)
M7 (n = 18)	1 (5.56)	17 (5.59)	1.000	0.950	1.069 (0.132–8.626)
Others (n = 42)	1 (5.56)	41 (13.49)	NA	NA	NA

Del, deletion.

For multiple comparisons, *p* value was set <0.05/2×(n−1) (n = 8).

a , non-cigarette smoking subjects who had no any history of cigarette smoking.

b , ORs (95% CIs) and *p* value determined by multivariate logistic regression analysis adjusted for age.

## Discussion

In the present study, mtDNA haplogroups D and F were found to be beneficial for individuals' resistance to lung cancer, whereas haplogroups G and M7 were associated with an increased risk for lung cancer in a Han Chinese population from southwestern China. In addition, cigarette smoking was a risk factor for the 822 bp mtDNA deletion and mtDNA haplogroups D was susceptible to damage from external ROS caused by cigarette smoking. The findings provided evidences from the point of mtDNA that the gene-environment interaction does exist in the individual susceptibility to lung cancer. To our knowledge, this is the first study to report the association of lung cancer risk with the mtDNA variations and of the cigarette smoking with the 822 bp mtDNA deletion that begins between 15587–15591 nps and ends between 16408–16412 nps.

The 822 bp mtDNA deletion was accidentally found to be present in many samples when the primers mtDNA-1 and mtDNA-2 were used to amplify the mtDNA fragment for subsequent sequencing of HVS I in the study. In principle, by using the nested-PCR protocol, it is possible to concentrate deleted molecules at the first PCR step and detect single molecules. Thus, all samples (cases and controls) were then re-amplified using the more sensitive nested-PCR method to detect extremely low levels of the mtDNA deletion. To investigate the role of the mtDNA deletion in the study population, the frequencies of mtDNA deletion were compared between light cigarette smoking subjects and heavy cigarette smoking subjects of combined cases and controls. The comparison revealed that the mtDNA deletion was significantly more frequent in heavy smoking subjects compared with light smoking subjects. Multivariate logistic regression analysis revealed that cigarette smoking was a risk factor for the mtDNA deletion. Many of the substances in cigarette smoke are chemicals that may create ROS within human body to introduce oxidative stress which was thought to be involved in lung carcinogenesis [37], [38] and mtDNA mutations [39]–[41]. Recently, it has been reported that the increased mtDNA copy number was associated with future development of lung cancer among heavy smokers for compensation for damage due to limited DNA repair capacity of mitochondrial [26]. Thus, the prior findings that heavy cigarette smokers would have higher internal doses of ROS [42], [43] and increased mtDNA copy number [26] than lighter cigarette smokers supported our findings that heavy smokers would have increased frequencies of the mtDNA deletion compared with light cigarette smokers.

MtDNA deletions are generally believed to be the results of oxyradical-induced DNA damage, but the mechanism of deletion is poorly understood. Most mtDNA deletions are predominantly (∼85%) flanked by short direct repeats [44], [45]. Presently, there are two proposals about the formation of mtDNA deletions. One proposal is that mtDNA deletion could be generated through a slipped-stand replication mechanism [46]. But the proposal was challenged by the recent modification of the strand-displacement model which argues that large regions of single-stranded DNA do not exist, rather, the lagging-strand template is largely protected by RNA [47], [48]. Another proposal is that mtDNA deletions could be generated during repair of damaged DNA caused by increased oxidative stress from whatever causes [49]. The later proposal was supported by many evidences from *E. coli*, mice to human cells [50]–[53]. The 822 bp mtDNA deletion detected in the study is also flanked by 5 bp short direct repeats (CTCCG) (Fig. 1C). Our findings provided indirect evidence to support the later proposal that the mtDNA deletions could be created during repair of damaged DNA generated by cigarette smoking. Understanding the mechanism involved in generation and subsequent clonal expansion is worthwhile to investigate further.

Compared with controls, the mtDNA deletion was found to be enriched in cases. In order to investigate whether the mtDNA deletion was associated with some mtDNA haplogroups, the frequencies of the mtDNA deletion in major mtDNA haplogroups of combined cases and controls were analyzed and the data revealed that the deletion occurred significantly more frequently in mtDNA haplogroups D and multivariate logistic regression analyses revealed that haplogroup D might be a risk factor for the mtDNA deletion. Since the cigarette smoking was a risk factor for the mtDNA deletion and no female cigarette smoking subjects were gathered in the study, the male subjects pooled from cases and controls were stratified by cigarette smoking into male cigarette smoking subjects and male non-cigarette smoking subjects to analyze further the association of mtDNA deletion with mtDNA haplogroups. The analysis of the frequencies of the mtDNA deletion in major mtDNA haplogroups among male cigarette smoking subjects also revealed that haplogroup D might be risk factors for the mtDNA deletion. No similar significant difference was found among male non-cigarette smoking subjects. However, mtDNA haplogroup G was found to be a risk factor for the deletion among male non-cigarette smoking subjects. Interestingly, the deletion was enriched in female non-cigarette smoking subjects compared with male non-cigarette smoking subjects of combined cases and control. One reason we hypothesized to explain this difference is that cooking oil fumes could be a greater risk factor for the mtDNA deletion for females who cook far more often than men in southwestern China.

Several mtDNA haplogroups were found to play roles in human longevity, carcinogenesis, Leber's hereditary optic neuropathy (LHON), and other metabolic and degenerative diseases. MtDNA haplogroup D is one of these mtDNA haplogroups. Haplogroup D is defined by the specific variation C5178A in mitochondrial NADH dehydrogenase subunit 2 (ND2). Prior studies showed that the protective effect of a Leu→Met substitution at amino acid 237 (L237M) of ND2 (C5178A) against oxidative damage to mitochondria not only contributes to human longevity [13], [16], but also provides strong anti-atherosclerotic effects in diabetic patients and protects against myocardial infarction [14]. Thus, the protective effect of haplogroup D against oxidative damage might also decrease the risk of lung cancer. The frequency of haplogroup J, which was found to be correlated with lower efficiency of the electron transport chain (ETC), diminished ATP, diminished ROS production, and accumulation in elderly people, was increased in patients with LHON and multiple sclerosis due to its limited power to compensate the mitochondrial energetic deficiency [18], [19]. Similarly, haplogroup D could account for the increased frequency of the C5178A variant in elderly people, which is caused by decreased oxidative damage [13], [16]. However, once the external ROS created by cigarette smoking increases, the frequency of the mtDNA deletion increases in cigarette-smoking subjects with haplogroup D. One reason we hypothesized to explain the phenomena that the mtDNA haplogroup D was found to be protective in lung cancer while the mtDNA deletion was enriched in male cigarette-smoking subjects with mtDNA haplogroup D of combined cases and controls is that individuals with haplogroup D might be susceptible to damage of external ROS caused by cigarette smoking. The methionine residues have been proposed to constitute an important anti-oxidant defense mechanism [54]. According to the predicted model of human ND2 molecular [55], the Leu→Met substitution at amino acid 237 (L237M) of ND2 (C5178A) is exposed at the surface of complex I and may play important roles in protective effect against oxidative damage to mitochondria as an efficient oxidant scavenger [13]. Localization of the methionine residue (Leu→Met) on the surface of the complex I might also be a target for damage of increased ROS whatever caused. The damaged residue or surface structure of the complex I might cause or exacerbate the deletion of mtDNA. The other reason we supposed to explain the phenomena is that the lung cancer risk and the mtDNA deletion were not influenced solely by the mtDNA variations. The nuclear background in which the mtDNA haplogroups are classified and the mtDNA deletion was found may also contribute to the individuals' susceptibility to lung cancer risk and the mtDNA deletion. Investigating the generation of endogenous ROS and the protective effect against oxidative damage to mitochondria under different conditions of different mtDNA haplogroups with the same nuclear background, or investigating the different nuclear background with the same mtDNA haplogroups, may help us, at least in part, to explore the fundamental mechanisms.

Haplogroup F was also identified less often in lung cancer cases as haplogroup D, but no significant difference was found in the frequencies of the mtDNA deletion among male cigarette-smoking subjects with haplogroup F. Haplogroup F has been divided into sub-haplogroups F1–4 in East Asia [35]. We speculate that haplogroup F might confer decreased lung cancer risk through some antioxidant defense mechanism. It is worthwhile to investigate further the role of haplogroup F in lung cancer resistance.

Both haplogroups M7 and G were found to be risk factors for lung cancer in this study. Haplogroup M7 was defined by a specific variation at np T9824C [34] and Haplogroup G was classified with the presence of the combined RFLP-4830 *Hae*II site/+4831 *Hha*I site [56]. Previous studies have demonstrated that M7b1'2, a sub-haplogroup of M7, was found to increase the penetrance of LHON [57] and haplogroup M7 was a risk factor for mild acute mountain sickness (AMS) [27]. In the present study, haplogroups G and M7 were found to be present in controls at frequencies of 2.78% and 5.95%, respectively, but were present in cases of lung cancer at frequencies of 7.92% and 12.67%, respectively. The findings that no significant difference was observed in the frequencies of the mtDNA deletion in male cigarette smoking subjects with haplogroup M7 or G indicated that the two haplogroups might better tolerate the damage of external ROS caused by cigarette smoking due to their relatively higher endogenous ROS production, which was supported by the subsequent finding that the frequencies of the mtDNA deletion increased in male non-cigarette smoking subjects with haplogroup G.

Though we conducted a population-based case-control study, there were several limitations. First, due to limited cases, we did not analyze the distribution of the mtDNA haplogroups or the mtDNA deletion in each type of lung cancer to investigate whether the risk of a certain type of lung cancer was associated with one or more mtDNA haplogroups or with the mtDNA deletion. Second, because the distribution of mtDNA haplogroups varies spatially in China, large-scale studies are required to clarify the association of mtDNA variation with lung cancer risk in other populations.

In summary, we conducted a case-control study to investigate the role of mtDNA variations in individual risk of lung cancer in a Han Chinese population from southwestern China. Our findings suggested that mtDNA haplogroups G and M7 might be risk factors for lung cancer, whereas haplogroups D and F might decrease the risk of lung cancer in the study population. In addition, the study revealed that mtDNA haplogroup D might be relatively more susceptible to DNA damage from the external ROS caused by cumulative cigarette smoking. And the 822 bp mtDNA deletion, which was positively associated with cigarette smoking, would be a potential biomarker for the exogenous and endogenous exposures that are associated with subsequent cigarette-smoking-related diseases.

## References

[1] Zhi XY (2009a). Advances in surgical treatment of non-small cell lung cancer.. Oncology Progress.

[2] Zhi XY (2009b). Epidemiological analysis of lung cancer in China.. Chinese Prescription Drugs.

[3] Schabath MB, Spitz MR, Hong WK, Delclos GL, Reynolds WF (2002). A myeloperoxidase polymorphism associated with reduced risk of lung cancer.. Lung cancer.

[4] Kiyohara C, Yoshimasu K, Shirakawa T, Hopkin JM (2004). Genetic polymorphisms and environmental risk of lung cancer: a review.. Rev Environ Health.

[5] Cote ML, Chen W, Smith DW, Benhamou S, Bouchardy C (2009). Meta- and pooled analysis of GSTP1 polymorphism and lung cancer: a HuGE-GSEC review.. Am J Epidemiol.

[6] De Ruyck K, Szaumkessel M, De Rudder I, Dehoorne A, Vral A (2007). Polymorphisms in base-excision repair and nucleotide-excision repair genes in relation to lung cancer risk.. Mutat Res.

[7] Wang Y, Yang H, Li L, Wang H, Zhang C (2010). Association between CYP2E1 genetic polymorphisms and lung cancer risk: a meta-analysis.. Eur J Cancer.

[8] Chen Z, Li Z, Niu X, Ye X, Yu Y (2011). The effect of CYP1A1 polymorphisms on the risk of lung cancer: a global meta-analysis based on 71 case-control studies.. Mutagenesis.

[9] Rafnar T, Sulem P, Besenbacher S, Gudbjartsson DF, Zanon C (2011). Genome-wide significant association between a sequence variant at 15q15.2 and lung cancer risk.. Cancer Res.

[10] Wallace DC, Ye JH, Neckelmann SN, Singh G, Webster KA (1987). Sequence analysis of cDNAs for the human and bovine ATP synthase beta subunit:mitochondrial DNA genes sustain seventeen times more mutations.. Curr Genet.

[11] Wallace DC, Fan W (2010). Energetics, epigenetics, mitochondrial genetics.. Mitochondrion.

[12] Tanaka M, Gong JS, Zhang J, Yoneda M, Yagi K (1998). Mitochondrial genotype associated with longevity.. Lancet.

[13] Takagi K, Yamada Y, Gong JS, Sone T, Yokota M (2004). Association of a 5178C→A (Leu237Met) polymorphism in the mitochondrial DNA with a low prevalence of myocardial infarction in Japanese individuals.. Atherosclerosis.

[14] Wallace DC (2005). A mitochondrial paradigm of metabolic and degenerative diseases, aging, and cancer: a dawn for evolutionary medicine.. Annu Rev Genet.

[15] Cai XY, Wang XF, Li SL, Qian J, Qian DG (2009). Association of mitochondrial DNA haplogroups with exceptional longevity in a Chinese population.. PLoS ONE.

[16] Moreno-Loshuertos R, Acín-Pérez R, Fernández-Silva P, Movilla N, Pérez-Martos A (2006). Differences in reactive oxygen species production explain the phenotypes associated with common mouse mitochondrial DNA variants.. Nat Genet.

[17] Marcuello A, Martínez-Redondo D, Dahmani Y, Terreros JL, Aragonés T (2009a). Steady exercise removes VO(2max) difference between mitochondrial genomic variants.. Mitochondrion.

[18] Marcuello A, Martínez-Redondo D, Dahmani Y, Casajús JA, Ruiz-Pesini E (2009b). Human mitochondrial variants influence on oxygen consumption.. Mitochondrion.

[19] Martínez-Redondo D, Marcuello A, Casajús JA, Ara I, Dahmani Y (2010). Human mitochondrial haplogroup H: the highest VO2max consumer—is it a paradox?. Mitochondrion.

[20] Croteau DL, Bohr VA (1997). Repair of oxidative damage to nuclear and mitochondrial DNA in mammalian cells.. J Biol Chem.

[21] Wallace DC (1999). Mitochondrial diseases in man and mouse.. Science.

[22] Church DF, Pryor WA (1985). Free-radical chemistry of cigarette smoke and its toxicological implications.. Environ Health Perspect.

[23] Pryor WA, Stone K (1993). Oxidants in cigarette smoke. Radicals, hydrogen peroxide, peroxynitrate, and peroxynitrite.. Ann N Y Acad Sci.

[24] Lee HC, Yin PH, Lin JC, Wu CC, Chen CY (2005). Mitochondrial genome instability and mtDNA depletion in human cancers.. Ann N Y Acad Sci.

[25] Yu M, Shi Y, Wei X, Yang Y, Zang F (2009). Mitochondrial DNA depletion promotes impaired oxidative status and adaptive resistance to apoptosis in T47D breast cancer cells.. Eur J Cancer Prev.

[26] Hosgood HD, Liu CS, Rothman N, Weinstein SJ, Bonner MR (2010). Mitochondrial DNA copy number and lung cancer risk in a prospective cohort study.. Carcinogenesis.

[27] Li FX, Ji FY, Zheng SZ, Yao W, Xiao ZL (2011). MtDNA haplogroups M7 and B in southwestern Han Chinese at risk for acute mountain sickness.. Mitochondrion.

[28] Torroni A, Lott MT, Cabell MF, Chen YS, Lavergne L (1994). MtDNA and the origin of Caucasians. identification of ancient Caucasian-specific haplogroups, one of which is prone to a recurrent somatic duplication in the D-loop region.. Am J Hum Genet.

[29] Starikovskaya EB, Sukernik RI, Derbeneva OA, Volodko NV, Ruiz-Pesini E (2005). Mitochondrial DNA diversity in indigenous populations of the southern extent of Siberia, and the origins of Native American haplogroups.. Ann Hum Genet.

[30] Andrews RM, Kubacka I, Chinnery PF, Lightowlers RN, Turnbull DM (1999). Reanalysis and revision of the Cambridge reference sequence for human mitochondrial DNA.. Nat Genet.

[31] Saillard J, Forster P, Lynnerup N, Bandelt HJ, Nørby S (2000). MtDNA variation among Greenland Eskimos: the edge of the Beringian expansion.. Am J Hum Genet.

[32] Derbeneva OA, Starikovskaya EB, Wallace DC, Sukernik RI (2002a). Traces of early Eurasians in the Mansi of northwest Siberia revealed by mitochondrial DNA analysis.. Am J Hum Genet.

[33] Derbeneva OA, Sukernik RI, Volodko NV, Hosseini SH, Lott MT (2002b). Analysis of mitochondrial DNA diversity in the Aleuts of the Commander Islands and its implications for the genetic history of Beringia.. Am J Hum Genet.

[34] Yao YG, Kong QP, Bandelt HJ, Kivisild T, Zhang YP (2002). Phylogeographic differentiation of mitochondrial DNA in Han Chinese.. Am J Hum Genet.

[35] Yao YG, Kong QP, Wang CY, Zhu CL, Zhang YP (2004). Different matrilineal contributions to genetic structure of ethnic groups in the Silk Road region in China.. Mol Biol Evol.

[36] Mishmar D, Ruiz-Pesini E, Golik P, Macaulay V, Clark AG (2003). Natural selection shaped regional mtDNA variation in humans.. Proc Natl Acad Sci USA.

[37] Hecht SS (1999). Tobacco smoke carcinogens and lung cancer.. J Natl Cancer Inst.

[38] Gackowski D, Speina E, Zielinska M, Kowalewski J, Rozalski R (2003). Products of oxidative DNA damage and repair as possible biomarkers of susceptibility to lung cancer.. Cancer Res.

[39] Vallyathan V, Shi X (1997). The role of oxygen free radicals in occupational and environmental lung diseases.. Environ Health Perspect.

[40] Cakir Y, Yang Z, Knight CA, Pompilius M, Westbrook D (2007). Effect of alcohol and tobacco smoke on mtDNA damage and atherogenesis.. Free Radic Biol Med.

[41] Ballinger SW, Bouder TG, Davis GS, Judice SA, Nicklas JA (1996). Mitochondrial genome damage associated with cigarette smoking.. Cancer Res.

[42] Morrow JD, Frei B, Longmire AW, Gaziano JM, Lynch SM (1995). Increase in circulating products of lipid peroxidation (F2-isoprostanes) in smokers. Smoking as a cause of oxidative damage.. N Engl J Med.

[43] Asami S, Hirano T, Yamaguchi R, Tomioka Y, Itoh H (1996). Increase of a type of oxidative DNA damage, 8-hydroxyguanine, and its repair activity in human leukocytes by cigarette smoking.. Cancer Res.

[44] Bua E, Johnson J, Herbst A, Delong B, McKenzie D (2006). Mitochondrial DNA-deletion mutations accumulate intracellularly to detrimental levels in aged human skeletal muscle fibers.. Am J Hum Genet.

[45] Samuels DC, Schon EA, Chinnery PF (2004). Two direct repeats cause most human mtDNA deletions.. Trends Genet.

[46] Shoffner JM, Lott MT, Voljavec AS, Soueidan SA, Costigan DA (1989). Spontaneous Kearns-Sayre/chronic external ophthalmoplegia plus syndrome associated with a mitochondrial DNA deletion: a slip-replication model and metabolic therapy.. Proc Natl Acad Sci USA.

[47] Yasukawa T, Reyes A, Cluett TJ, Yang MY, Bowmaker M (2006). Replication of vertebrate mitochondrial DNA entails transient ribonucleotide incorporation throughout the lagging strand.. EMBO J.

[48] Yang MY, Bowmaker M, Reyes A, Vergani L, Angeli P (2002). Biased incorporation of ribonucleotides on the mitochondrial L-strand accounts for apparent strand-asymmetric DNA replication.. Cell.

[49] Krishnan KJ, Reeve AK, Samuels DC, Chinnery PF, Blackwood JK (2008). What causes mitochondrial DNA deletions in human cells?. Nat Genet.

[50] Horiguchi M, Masumura KI, Ikehata H, Ono T, Kanke Y (2001). Molecular nature of ultraviolet B light-induced deletions in the murine epidermis.. Cancer Res.

[51] Sargentini NJ, Smith KC (1992). Involvement of RecB-mediated (but not RecF mediated) repair of DNA double-strand breaks in the gamma-radiation production of long deletions in Escherichia coli.. Mutat Res.

[52] Thacker J, Chalk J, Ganesh A, North P (1992). A mechanism for deletion formation in DNA by human cell extracts: the involvement of short sequence repeats.. Nucleic Acids Res.

[53] Esposito LA, Melov S, Panov A, Cottrell BA, Wallace DC (1999). Mitochondrial disease in mouse results in increased oxidative stress.. Proc Natl Acad Sci USA.

[54] Levine RL, Mosoni L, Berlett BS, Stadtman ER (1996). Methionine residues as endogenous antioxidants in proteins.. Proc Natl Acad Sci USA.

[55] Hirokawa T, Seah BC, Mitaku S (1998). SOSUI: classification and secondary structure prediction system for membrane proteins.. Bioinformatics.

[56] Kivisild T, Shen P, Wall DP, Do B, Sung R (2006). The role of selection in the evolution of human mitochondrial genomes.. Genetics.

[57] Ji Y, Zhang AM, Jia X, Zhang YP, Xiao X (2008). Mitochondrial DNA haplogroups M7b1'2 and M8a affect clinical expression of leber hereditary optic neuropathy in Chinese families with the m.11778G→a mutation.. Am J Hum Genet.

